# Micromechanical Loading Studies in Ex Vivo Cultured Embryonic Rat Bones Enabled by a Newly Developed Portable Loading Device

**DOI:** 10.1007/s10439-023-03258-2

**Published:** 2023-06-14

**Authors:** Zhengpei Zhang, Farasat Zaman, Tobia Sebastiano Nava, Tim R. J. Aeppli, Elena M. Gutierrez-Farewik, Artem Kulachenko, Lars Sävendahl

**Affiliations:** 1 Division of Pediatric Endocrinology, Department of Women’s and Children’s Health, Karolinska Institutet, Solna, Sweden; 2 KTH MoveAbility Lab, Department of Engineering Mechanics, KTH Royal Institute of Technology, Stockholm, Sweden; 3 Solid Mechanics Unit, Department of Engineering Mechanics, KTH Royal Institute of Technology, Stockholm, Sweden

**Keywords:** Bone growth, Dynamic load, Bone organ culture, Metatarsal, Femur

## Abstract

Mechanical loading has been described as having the potential to affect bone growth. In order to experimentally study the potential clinical applications of mechanical loading as a novel treatment to locally modulate bone growth, there is a need to develop a portable mechanical loading device enabling studies in small bones. Existing devices are bulky and challenging to transfer within and between laboratories and animal facilities, and they do not offer user-friendly mechanical testing across both ex vivo cultured small bones and in vivo animal models. To address this, we developed a portable loading device comprised of a linear actuator fixed within a stainless-steel frame equipped with suitable structures and interfaces. The actuator, along with the supplied control system, can achieve high-precision force control within the desired force and frequency range, allowing various load application scenarios. To validate the functionality of this new device, proof-of-concept studies were performed in ex vivo cultured rat bones of varying sizes. First, very small fetal metatarsal bones were microdissected and exposed to 0.4 N loading applied at 0.77 Hz for 30 s. When bone lengths were measured after 5 days in culture, loaded bones had grown less than unloaded controls (*p* < 0.05). Next, fetal rat femur bones were periodically exposed to 0.4 N loading at 0.77 Hz while being cultured ex vivo for 12 days. Interestingly, this loading regimen had the opposite effect on bone growth, i.e., loaded femur bones grew significantly more than unloaded controls (*p* < 0.001). These findings suggest that complex relationships between longitudinal bone growth and mechanical loading can be determined using this device. We conclude that our new portable mechanical loading device allows experimental studies in small bones of varying sizes, which may facilitate further preclinical studies exploring the potential clinical applications of mechanical loading.

## Introduction

Longitudinal bone growth occurs in the epiphyseal growth plates, wherein chondrocytes undergo a slow division process and then rapidly proliferate and enlarge [1, 2]. The growth process is tightly regulated by numerous factors [3], including genes [4], systemic factors [5], local factors [6], and mechanical loading [7].

Ex vivo bone cultures provide a unique platform for experimental bone research [8]. They simplify numerous complexities of in vivo animal experiments and allow well-controlled experimental settings so that various factors, such as mechanical loading, can be investigated independently. The effects of mechanical loading on ex vivo cultured bones have been investigated in numerous studies [8]. Ex vivo cultured fetal metatarsal bones are a well-established model for studying longitudinal bone growth and responsiveness to different growth-regulating factors, including insulin-like growth factor-I (IGF-1), glucocorticoids and radial shock waves [9, 10]. Additionally, the fetal femur bone has a high density of growth plate chondrocytes, as visualized by histological staining, thus providing great potential for ex vivo studies of longitudinal bone growth [11].

The effects of mechanical loading on longitudinal bone growth were described by Hueter–Volkmann, who found that that increased mechanical compression retards bone growth. In contrast, lower-than-normal loading accelerates bone growth [12]. The types of mechanical loading that have been applied to ex vivo cultured bone organs include compression [13], perfusion [14], hydrostatic pressure [15], vibration [16], stretch [17], three-point bending [18], the Zetos culture loading system [19] and unloading [20]. However, to the best of our knowledge, no studies have explored the effects of mechanical loading characteristics on bone growth in cultured explant embryonic metatarsal and femur bones.

One of the largest challenges in mechanobiology studies of small ex vivo models is the lack of microloading devices; the numerous mechanical loading test devices presently available on the market have limited feasibility in this application, as they are generally large, heavy, and difficult to move between laboratories and animal facilities. One such test device is the ElectroForce loading test instrument (1051 × 579 × 522 mm), which can deliver a wide range of mechanical forces and has been used to investigate the effects of loading in bladder organ tissue [21]. Other devices include the Marshall compression testing machine (500 × 450 × 1400 mm) and the tensile and compression testing machine from MinebeaMitsumi (637 × 1592 × 503 mm). All existing devices lack a frame, which would make it possible to implement a simplified and straightforward mechanical loading protocol in both in vitro and in vivo study designs.

To address the need for a portable mechanical loading device, our primary aim was to develop a small device that is easy to operate and can apply dynamic mechanical loading with a controller and a frame, thereby useful for protocols with varying load, frequency, and treatment duration. Our secondary aim was to perform proof-of-concept experiments, in which we studied this device’s efficacy in investigating the effects of micromechanical loading on longitudinal bone growth in small embryonic rat metatarsal and femur bones measuring 1 mm and 4 mm in length, respectively.

## Materials and Methods

### Inventory List

#### Main Components

- Linear Actuator V-275.431 (Physik Instrumente, Germany),
- Actuator Controller C-413.1G (Physik Instrumente, Germany),
- Laptop 20HF-004UMX S/N PC-OSX4ZY 18/02 (Lenovo, China),
- Wireless Broadband Router GN-BR01G (Gigabyte, China),
- Mounting Frame, in-house manufacturing.

#### Loading Instruments

- Indenter with convex elastomer cover (Physik Instrumente, Germany),
- Flat indenter without cover, in-house manufacturing,
- 3D-printed needle attachments, in-house manufacturing,
- Customized sewing machine needle bar, Singer 257.

### Design of the Microloading Device

The microloading device was designed to facilitate loading in both ex vivo cultured bones and in vivo conditions and reduced the chances for damage of the samples. Specifically, two use-cases were devised: repetitive impact loading of small force (0.05–0.5 N) at a range of  30–180 repetitions and continuous sinusoidal loading of medium force (0.5–5 N) at a frequency range of 5–20 Hz. In addition, the device was developed to have exchangeable tools and the ability to adjust the height of the exchangeable tools of a position of minimally 80 mm to allow proper positioning and adjusting sample, which gave the user flexibility and ease of setup and minimize risk of sample damage.

#### Design Working Principle

The actuator was embedded into a stainless-steel frame, providing the essential framework and interfaces. It provides the required sensory output, such as applied force and position. A working platform above the actuator protects the actuator from potential damage and makes it possible for the user to easily place the samples. It also has a detachable cover for accessibility to exchange the lower tool.

**Fig. 1:**
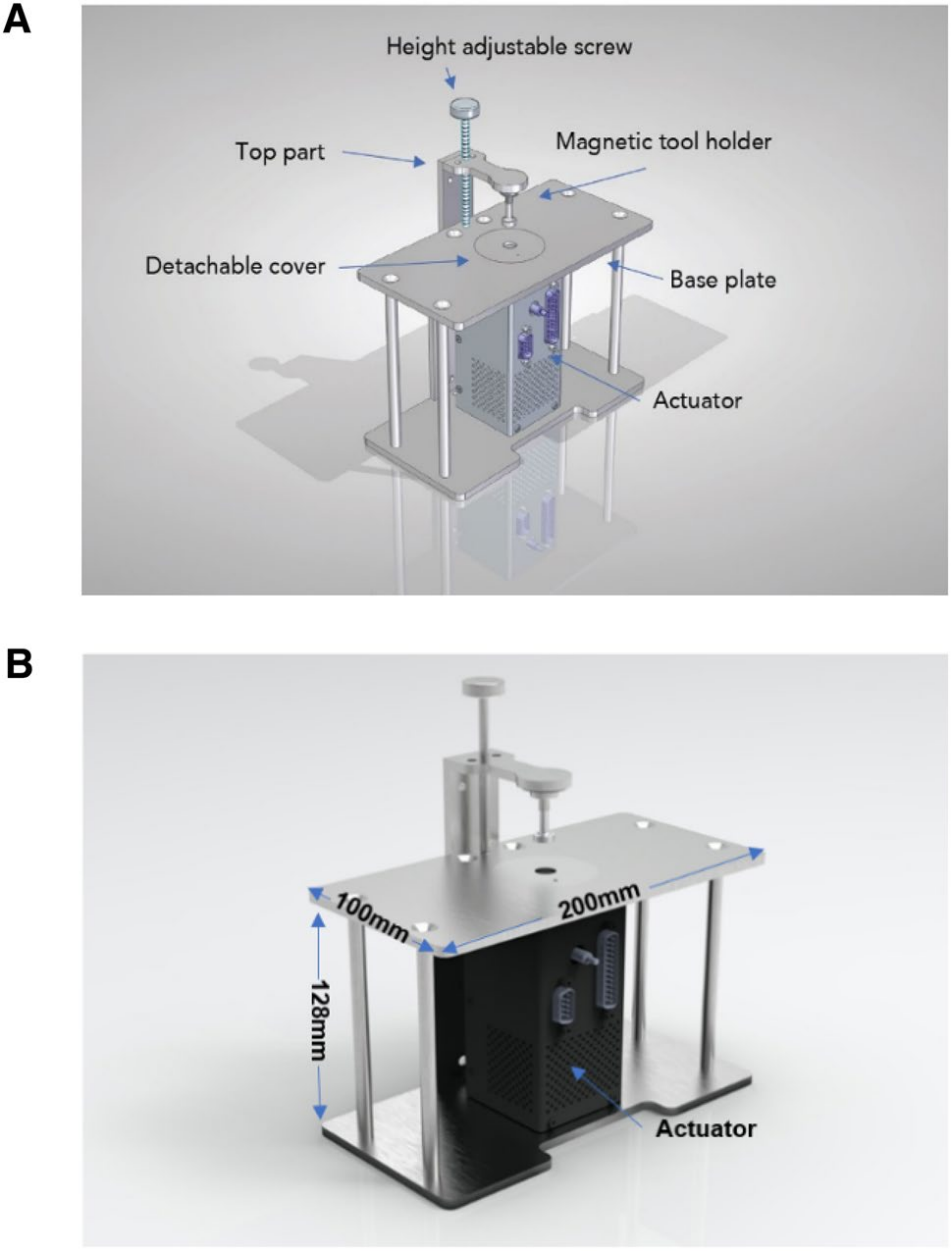
Microloading device. **A** Design of the microloading device with individual parts annotated. **B** Complete system dimensions: 200 mm width, 128 mm height, and 100 mm depth

The height-adjustable screw and top part can be adjusted for suitable sample heights using butterfly nuts and precise locking. All individual components of the system are visualized in Fig. 1A. The completed design measures 200 mm × 128 mm × 100 mm, as shown in Fig. 1B.

### Operation of the Microloading Device

#### System Connection Setup

**Fig. 2:**
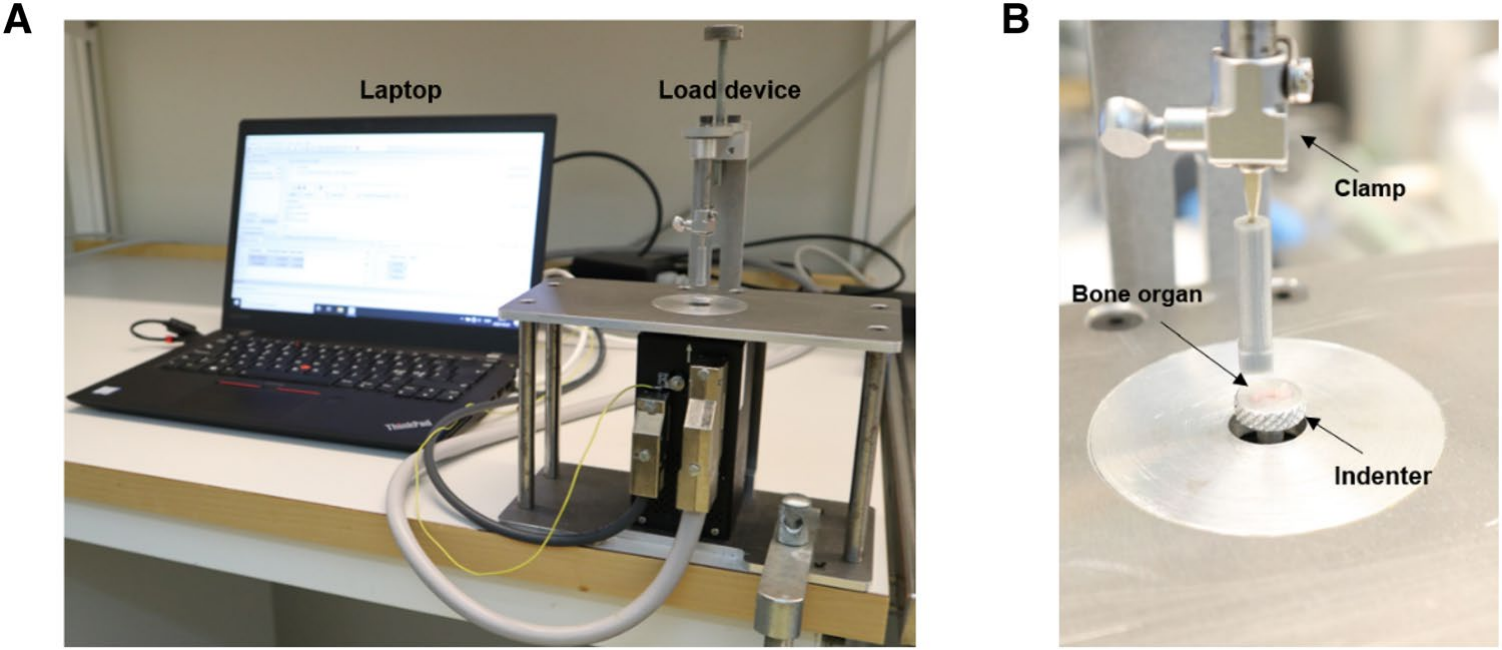
System setup. **A** Microloading device connected to a laptop computer. **B** Adjustment of the ex vivo bone organ tool

The microloading device was clamped to the table securely and connected to the laptop (Fig. 2A), controller, and router. The PIMikroMove program was used to control the actuator, which was set at a reference position (0 mm). An ex vivo bone testing tool involves a clamp in which one can insert a needle covered with different 3D-printed attachments. The magnetic interface to the top section allows a simple assembly as well as fine adjustment to orient the tool in relation to the bone samples. The actuator holds the indenter and applies force to the bone sample. The tool to support bone samples can be adjusted to a suitable distance from the indenter using the height-adjusting screw (Fig. 2B).

### Built-in Force Recording System

A router is required for communication between the controller and the computer. Using the software framework provided by the actuator manufacturer (PIMikroMove 2.29.7.0, Solid Edge 2019), custom macro-scripts were written for the individual application cases. This enables process automation and loading repeatability. The macro-script can be structured to incorporate all relevant parameters, such as load frequency, duration, amplitude, and preloading.

### Fujifilm Prescale® Pressure Indicating Sensor Film

Fujifilm Prescale® (Japan) was used to verify the load applied by the microloading device. This pressure-sensitive film changes color at intensities proportional to pressure magnitude. It is capable of determining pressures from 0.1 MPa. To verify applied pressure, Fujifilm Prescale® was placed under an 8-week-old mouse femur bone while forces with a range of amplitudes were applied, including 0.05 N, 0.25 N, 0.5 N, 1 N, 2 N, 3 N, 4 N, 5 N, 6 N, and 7 N.

### Ex Vivo Bone Culture System

**Fig. 3:**
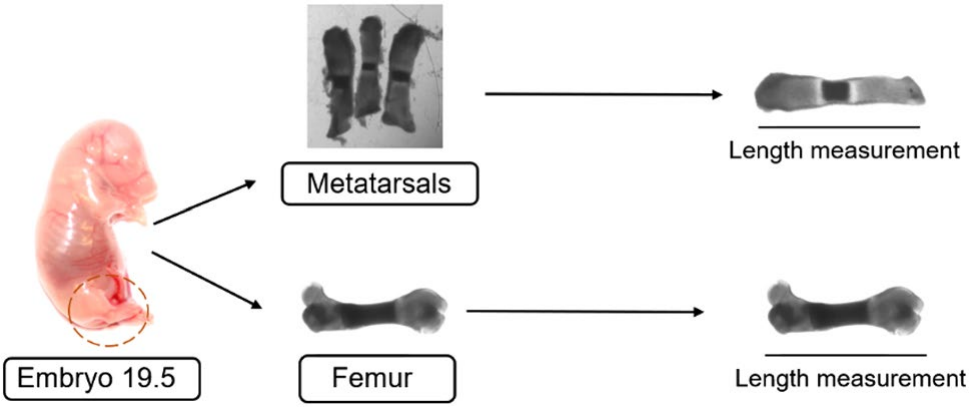
Scheme for embryonic bone dissection

All ex vivo bone experiments were approved by the local ethical committee (Stockholm, North Animal Ethics Committee, Sweden). The three middle metatarsal bones and one femur bone were dissected from each hind limb of rat embryos on Day 19.5 of gestation (Fig. 3). After dissection, each bone was transferred to a 24-well plate and cultured in minimum essential medium α (MEM-α, Invitrogen, Scotland) supplemented with 0.2% endotoxin-free BSA, 1 mM β-glycerophosphate, 0.05 mg/ml ascorbic acid and 20 μg/ml gentamycin at 37 °C under a humidified atmosphere with 5% CO_2_ [10]. The medium was changed every 2 to 3 days.

### Proof-of-Concept Tests on Metatarsal Bones

**Fig. 4:**
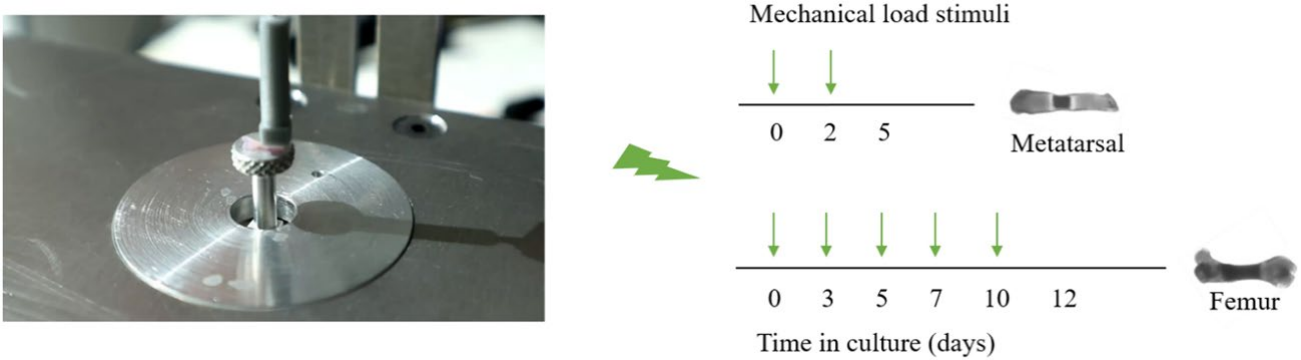
Micromechanical ex vivo loading of metatarsal and femur bones

The device was installed at room temperature and cleaned with 70% ethanol prior to bone loading. After excising metatarsal bones, they were randomly assigned into seven loaded groups and one control group. Loaded bones were placed back into the culture medium after loading. Control bones were placed on the indenter for the same time as the loaded bones but were not exposed to loading and then transferred back into the culture medium. Among the loaded metatarsal bones, a range of mechanical loading, specifically 0.1 N, 0.15 N, 0.2 N, 0.25 N, 0.3 N, 0.35 N, and 0.4 N at 0.77 Hz over 30 s, was applied on Days 0 and 2 after dissection (Fig. 4). IGF-1 (Sigma-Aldrich, Germany) at a concentration of 100 ng/ml is known to stimulate metatarsal bone growth and was used in the experiment as a positive control [22, 23]. The length of each bone was measured from images captured on Days 0, 2, and 5.

### Proof-of-Concept Tests on Femur Bones

Ex vivo cultured femur bones were similarly divided into two loaded groups and one control group and then placed in culture for 12 days. In the two loaded groups, 0.3 N and 0.4 N were applied at 0.77 Hz over 30 s on Days 0, 3, 5, 7, and 10 (Fig. 4). Control bones were, as described above, transferred to and from the device, but no load was applied. The lengths of all femur bones were measured from images captured on Days 0, 3, 5, 7, 10, and 12. For all tests, bone growth was expressed as the difference in bone length from Day 0 as a percent of size on Day 0.

### Statistics

Statistical analysis was carried out using GraphPad Prism 9 (USA). Bone growth data are presented as the mean percent increase in bone length ± SEM. One-way ANOVA was used to compare differences between groups, with the null hypothesis that growth was similar in all bone groups. A *p* value of < 0.05 was considered to indicate significance.

## Results

### Force Control Protocol

**Fig. 5:**
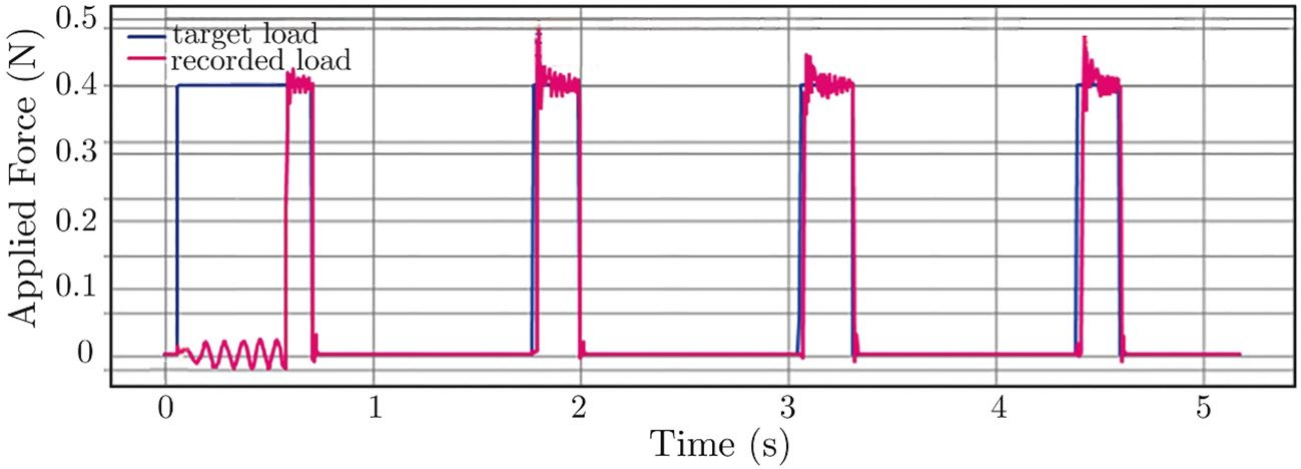
Example recording of a load of 0.4 N repetitively applied at a frequency of 0.77 Hz

The force control was verified by the software provided with the controller. Figure 5 shows the target and the recorded force input. The first cycle was used for the adjustments while ensuring that the maximum force was not surpassed, and consequent load cycles were repeatable and followed the target profile accurately with a reasonably low level of noise.

### Verification of Applied Load by a Pressure-Sensitive Film

**Fig. 6:**
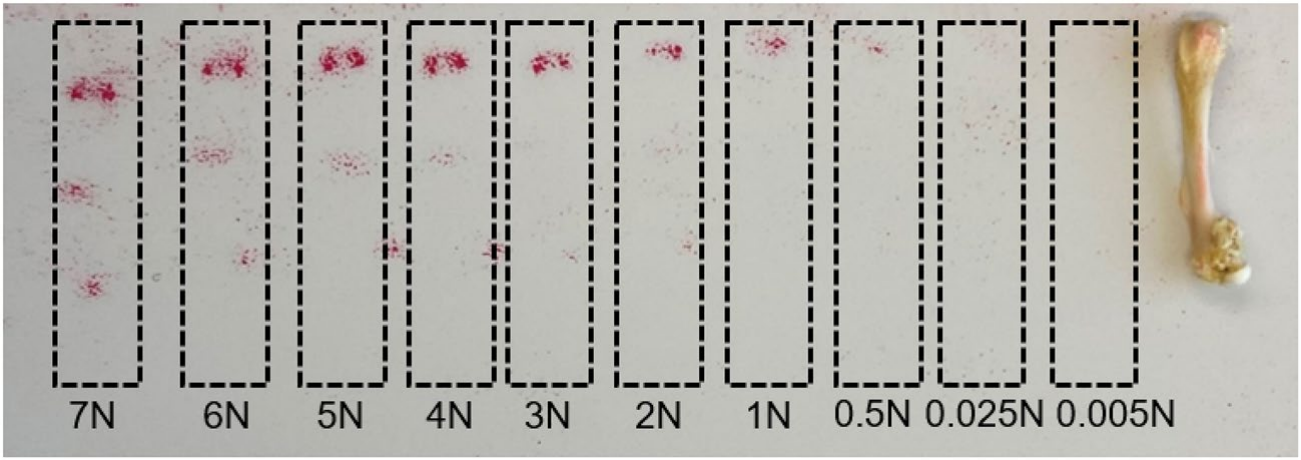
Load verification on an 8-week-old mouse femur loaded at a range of mechanical forces using pressure-sensitive Fujifilm Prescale®

The pressure range achieved by applying mechanical loading at different load levels was verified using the pressure-sensitive film. Figure 6 shows how the color intensity on the contact surface increases with increased load, indicating that the 0.1 MPa threshold is passed at approximately 0.5 N.

### Effect of Mechanical Loading on Bone Growth in Cultured Metatarsal Bones

**Fig. 7:**
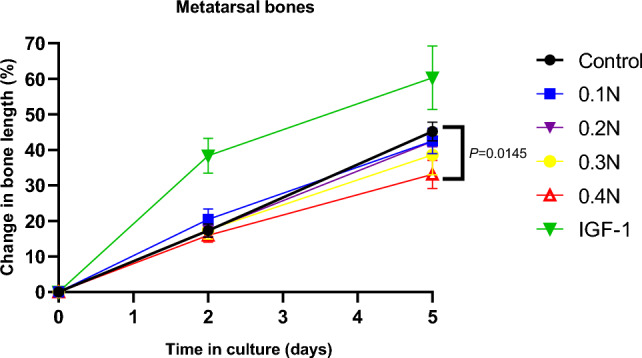
Dose-dependent effect of loading on the growth of ex vivo cultured metatarsal bones. Metatarsal bone growth was followed for five consecutive days in all groups (*n* = 4/group). In the loaded groups, metatarsal bones were subjected to mechanical loading of 0.1 N, 0.2 N, 0.3 N, and 0.4 N at 0.77 Hz over 30 s (0.15 N, 0.25 N, and 0.35 N are not shown in the figure). Means (represented by points) and SEMs (represented by error bars) are illustrated. One-way ANOVA indicates statistically different growth between the control and each loaded group is indicated

A dose-dependent suppression of metatarsal bone growth was found after 5 days of culture; bones loaded with 0.4 N grew significantly less than control bones (Fig. 7, *p* < 0.05).

### Effect of Mechanical Loading on Growth in Femur Bones Cultured Ex Vivo

**Fig. 8:**
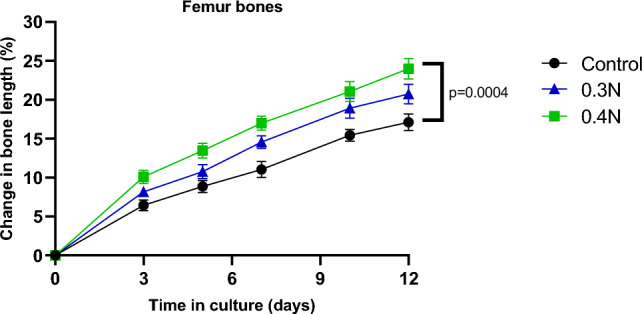
Dose-dependent loading effect on cultured femur bone growth ex vivo. Femur bone growth was followed for 12 consecutive days in 3 groups of bones (*n* = 6–18/group): loaded groups that were subjected to 0.3 N or 0.4 N for 30 s and a control group. Means (represented by points) and SEMs (represented by error bars) are illustrated

Mechanical loading at 0.3 N and 0.4 N (0.77 Hz) was applied over 30 s to the femur bones. Bones loaded at 0.4 N grew significantly more than control bones (*p* < 0.001, Fig. 8).

## Discussion

The first novel aspect of this study is the development of a microloading device that is small, easily constructed, and flexible, thus facilitating studies that examine the effects of mechanical loading in small ex vivo bone organs. This is in line with the 3R principles, reducing the number of animals needed for research purposes by maximizing the use of explant bone organs from one pregnant animal. Second, to the best of our knowledge, this is the first study to investigate longitudinal bone growth in fetal ex vivo femur bone organs. Third, the efficacy of the new device is supported by proof-of-concept experimental findings, which demonstrate growth suppression in small bones and stimulation in larger bones resulting from the same loading during the culture period. In fetal metatarsal bones, the highest tested mechanical loading (0.4 N at 0.77 Hz) was found to suppress bone growth. In contrast, the same load magnitude and frequency stimulated growth in the slightly larger fetal rat femurs. This is a unique finding that has never been reported before. This device will enable further systematic investigations with the aim of quantifying how loading and/or frequency affects growth in ex vivo cultured bones of different sizes, as well as studying whether pathological conditions alter this relationship. Furthermore, the device is dimensioned and equipped to enable similar experiments in vivo in small animals; the implemented force control protocol allows a precise load application in the time domain, which is vital for animal safety when used in vivo.

Based on these novel aspects, the device holds potential for various research applications:

(1) Pharmaceutical studies: for preclinical testing of drugs targeting bone growth and development, assuming that the chosen animal model is valid. (2) Veterinary medicine: to study bone growth in animals, helping veterinarians understand the effects of mechanical loading on various species' bones and contributing to improved treatment options for bone-related conditions in animals. (3) Biomedical research: in tissue engineering, biomechanics, and mechanobiology, to study the effects of mechanical loading on bone growth, deformity formation, and morphology. This can lead to new insights and discoveries in bone biology, ultimately contributing to the development of novel treatments and therapies.

In our experiments, we used a flat indenter to apply controlled loading to the curved surface of the growth plate in embryonic rat femur bones. Due to the geometry mismatch between the flat indenter and the curved bone surface, the contact interface load distribution may not be uniform. However, according to Saint-Venant's principle in solid mechanics, the stress and strain distributions become nearly independent of the specific loading conditions at distances sufficiently far from the load application region. In this study, our primary interest is understanding the bone tissue's overall response to the applied load. Therefore, we believe that the local surface effects near the contact region do not significantly impact the outcome of the loading as long as the applied force is the same. Furthermore, bone fixation and prevention of sample slippage are crucial but difficult to strictly control. To this end, we developed a customized 3D-printed needle attachment system for our ex vivo bone samples. In a series of pilot studies, we identified the ideal needle attachment size and shape (Fig. 2B) for securely holding the bone samples in place during the loading tests.

In this microloading device, the magnetic interface between the framework and the tools enables users to adjust positions horizontally, while the height-adjustable screw facilitates vertical adjustments. This design ensures an efficient pre-experimental setup and allows the user to accurately position the bone sample beneath the indenter. The 3D-printed needle attachment used during loading tests was determined through a series of pilot studies. Its square shape securely covers the entire cartilage area on each side of the embryonic femur bone, allowing the indenter to apply mechanical loading specifically to the growth plate in a stable manner. Another feature of this microloading device is the specially designed receptacle for small ex vivo bone organ samples. This receptacle ensures that the bone samples remain securely in place during the loading tests, preventing slippage and allowing for consistent compression in the same area throughout the experiment. The low variation in bone growth observed in our results indicates that the mechanical loading is repeatable, and the constraints are well-controlled.

Additional feature of this microloading device is the receptacle designed for small ex vivo bone organ samples. It does not apply any additional load to the bone samples due to, for instance, clamping, thereby facilitating precise alignment of the tool to deliver mechanical loading to the bone samples. The device's controller enables sinusoidal loading, which has been demonstrated to have a bone growth-promoting effect in mice [24]. Furthermore, as it is a force control device, it can prevent overloading bone samples or live animal specimens.

The position of the actuator at the bottom of the microloading device reduces the risk of inducing damage to the bone samples and the actuator. The actuator controls the indenter and induces the force during the loading process. As the range of motion and the associated accelerations are small, it can be assumed that inertial effects are negligible.

In this study, we identified that the same load applied to bones of different dimensions has opposing effects on bone growth, suggesting that the effects of mechanical loading on growth are dependent on the magnitudes and relative dimensions of the bones. Embryonic metatarsal bones from Day 19.5 of gestation are approximately 1 mm in length, whereas femur bones are approximately 4 mm, indicating that the level of mechanical loading that stimulates or inhibits bone growth is likely dependent on bone size. This could explain why contradictory data have been reported regarding how mechanical loading modulates longitudinal bone growth; some studies report mechanical suppression of bone growth [25–28], while others report stimulation [29–32]. Furthermore, we observed that a frequency as low as 0.77 Hz could stimulate bone growth compared to 20 Hz, as has been previously reported to stimulate bone growth in mice [24].

Our study has several limitations. First, the Fujifilm used in this study did not detect pressure with the lowest force magnitudes of 0.25 N and 0.05 N; these forces are below the film’s detection threshold. Second, this device cannot deliver a repetitive load at frequencies higher than 1 Hz. However, as most natural motions occur at equivalent frequencies of less than 1 Hz, it is possible to test a broad spectrum of loading frequencies that correspond to realistic motions. Last, even though the dimensions and features of the device are appropriate for in vivo studies in small animals, we have not investigated its efficacy in studying mechanical loading effects on bone growth in vivo*.* Further studies are warranted to test this device in live animals, with a wide range of mechanical loading levels, frequencies, and load durations.

Overall, this device can be used in mechanobiological studies in bones as small as 1 mm, in which it is possible to study direct effects on explant bone cultures. The opposing effects of loading on bone growth inspire further investigation in bone regenerative and biomaterial studies. For example, it could be of great interest to explore whether mechanical loading has the potential to be used as a new treatment in disorders where bone growth is affected, and local stimulation or inhibition of longitudinal bone growth is desired.

## Conclusion

The following contributions are made to the field of mechanobiology through the development of a portable microloading device specifically designed for small bones:

1. This device overcomes the feasibility limitations of existing devices, which are typically large and difficult to transport. The device's compact size, ease of construction, and portability make it highly suitable for various research settings. It has the potential to facilitate further mechanobiology studies by providing researchers with a user-friendly and portable device that is capable of applying dynamic mechanical loading to small bones.
2. The methodology employed is detailed and reproducible due to its relative simplicity and robustness, which was demonstrated in ex vivo bone testing. This ensures that other researchers can easily replicate the study and use the device in other experiments, thus expanding our knowledge in mechanobiology.
3. The results of the study demonstrate the effectiveness of the device. It was successfully tested using ex vivo cultured embryonic rat bones, wherein growth was influenced by loading magnitude. The study also investigated the effects of mechanical loading on bone growth in the embryonic metatarsal and femur bones ex vivo, illustrating that the relationship between load magnitude and bone dimensions are the key factors in the effect of mechanical loading on longitudinal bone growth. These findings contribute to a better understanding of the mechanobiological processes involved in bone growth and development, with potential implications for treating bone-related disorders.
